# Factors Affecting Salt Reduction Measure Adoption among Chinese Residents

**DOI:** 10.3390/ijerph18020445

**Published:** 2021-01-08

**Authors:** Zeying Huang, Di Zeng

**Affiliations:** 1 Institute of Food and Nutrition Development, Ministry of Agriculture and Rural Affairs, Beijing 100081, China; 2 Centre for Global Food and Resources, The University of Adelaide, Adelaide, SA 5005, Australia; di.zeng@adelaide.edu.au

**Keywords:** salt, reduction, diet, adoption, label, China

## Abstract

China has the highest mortality rate caused by diseases and conditions associated with its high-salt diet. Since 2016, China has initiated a national salt reduction campaign that aims at promoting the usage of salt information on food labels and salt-restriction spoons and reducing condiment and pickled food intake. However, factors affecting individuals’ decisions to adopt these salt reduction measures remain largely unknown. By comparing the performances of logistic regression, stepwise logistic regression, lasso logistic regression and adaptive lasso logistic regression, this study aims to fill this gap by analyzing the adoption behaviour of 1610 individuals from a nationally representative online survey. It was found that the practices were far from adopted and only 26.40%, 22.98%, 33.54% and 37.20% reported the adoption of labelled salt information, salt-restriction spoons, reduced condiment use in home cooking and reduced pickled food intake, respectively. Knowledge on salt, the perceived benefits of salt reduction, participation in nutrition education and training programs on sodium reduction were positively associated with using salt information labels. Adoption of the other measures was largely explained by people’s awareness of hypertension risks and taste preferences. It is therefore recommended that policy interventions should enhance Chinese individuals’ knowledge of salt, raise the awareness of the benefits associated with a low-salt diet and the risks associated with consuming excessive salt and reshape their taste choices.

## 1. Introduction

Over the past 40 years, people in China have been consuming, on average, 10 g of salt per day per person [1], which is twice as much as the internationally recommended intake [2]. The global mean salt intake would increase from 4.0 to 5.6 g/day if China was not included in the calculation [3]. Individuals should consume the amount of salt that is appropriate for their personal physiology [4], but high salt intake has been identified as an important behavioural cause of raised hypertension, cardiovascular disease and other diseases [5]. China has the highest mortality rate in the world caused by diseases associated with a high-salt diet [6]. Salt reduction is therefore of the utmost importance to cost-effectively combat these salt-induced health consequences [7].

China initiated a national salt reduction campaign in 2016, with the goal of reducing per capita salt intake to 5 g/day by 2030 [8]. To achieve this goal, *The Chinese Dietary Guidelines (2016)*, which are official dietary guidelines in China, recommend four salt reduction measures—reading salt information on food labels, using a salt-restriction spoon to measure a single salt dose, adding less condiments (i.e., condiments in this study are specified as soy sources and fish sources, although salt is one of the condiments) while cooking and reducing pickled food intake [9]. Chinese people are increasingly consuming processed foods (e.g., bacon, ham, salty snacks and instant noodles) [10].

In order to inform consumers of the exact amount of salt contained in foods and assist them in choosing products with less salt, *The General Rules for Nutrition Labeling of Pre-Packaged Food* make it mandatory for food industries in China only to label sodium content (i.e., milligram per serving or per 100 g/mL) on the packaging of all processed foods [11]. Salt-restriction spoons, particularly those measuring an amount of two grams, are utilized to calculate the amount of discretionary salt added in cooking or at the table. Such spoons, distributed for free by the Chinese government in recent years, have been proven to be effective in preventing cardiovascular diseases [12]. Moreover, condiments (e.g., soy sources and fish sources), commonly used to flavour food in China [13], account for 10.2% of total salt intake from home-cooked foods [14]. High-salt pickled foods (e.g., pickled vegetables, meat and seafood) are also traditionally consumed, usually together with porridge in China [15]. Reducing the intake of condiments and pickled foods thus plays an important role in achieving the goal of reducing salt consumption. Despite these effective salt reduction measures, it remains largely unclear how Chinese people adopt them and what the underlying factors are in shaping their adoption. Improved understanding of the adoption behaviour is therefore highly policy-relevant, as it could inform the design of policy interventions to incentivize the adoption and improve population health.

Existing studies on salt reduction practices mainly focus on labelling [10,16,17], while less attention has been paid to other practices, except for a few attempts to assess the usage of salt-restriction spoons [12,15], condiment use [18] and pickled food intake [14]. The particular focus on labelling stems from the fact that processed foods are the main source of dietary salt in developed countries [19]. In addition, there is no evidence that a specific salt reduction intervention is more effective than a suite of strategies combined [20], as few studies have assessed multiple practices in a comprehensive manner.

Previous studies have examined how the adoption of the above-mentioned salt reduction measures is influenced by: (1) sociodemographic characteristics such as gender [21], age [22] and education [23]; (2) economic conditions such as employment status [24] and income [24,25]; (3) living place [26]; and (4) health conditions such as weight status (overweight/obesity) [27]. In addition to these conventional factors, individual knowledge, attitudes and practices regarding salt intake are found to influence adoption, such as knowledge of salt consumption [28], awareness of hypertension [29], attitudes towards a low-salt diet [28,30], taste preference [30] and participation in nutrition education and sodium reduction training programs [31]. Adoption is also related to external/social resources, such as whether there is a community-based education program(s) on salt reduction [15] and whether food advertisements increase salt intake [32]. Family history of noncommunicable diseases, knowledge about healthy diets and dietary habits, as well as peers’ noncommunicable disease status also play their roles in stimulating salt reduction behaviour [12]. Furthermore, Zhang et al. [14] investigate the relationship between health status and pickled food intake and suggest that poor health is associated with greater pickled food consumption. How various factors, including those identified above, jointly affect the adoption of multiple salt reduction practices in China has not been investigated, a gap which our study seeks to fill.

The study employs a nationwide survey to explore the roles of a wide range of factors in shaping Chinese residents’ adoption of four key salt reduction measures, namely reading salt information on food labels, using a salt-restriction spoon, adding less condiments while cooking and reducing pickled food intake. Logistic regressions were used to detect/establish possible linkages between various factors and the four salt reduction measures, from which policy implications were drawn in order to promote their adoption in the wider population. To the best of our knowledge, this is the first study of its kind in China to empirically investigate the main salt reduction practices. The focus on China, the largest country by population, further sheds light on enhancing the preventive management of noncommunicable diseases and the effectiveness of salt reduction strategies that are already or soon to be implemented in other countries. This could contribute to achieving a higher welfare goal through healthy diet, disease prevention and public health intervention, at a global scale.

## 2. Materials and Methods

### 2.1. Methods

To compare the results from variable selection made by the various forms of logistic regression, the study sequentially applied logistic, stepwise logistic, lasso logistic and adaptive lasso logistic regressions. Model performances were carefully compared and evaluated to produce the best fit models and most robust results.

#### 2.1.1. Logistic Regression

This study started with a binary logistic regression model, a statistical method to predict a binary outcome using a collection of independent variables. The logistic function is expressed as follows:

(1) $$y_{i}=\ln\left[\frac{prob\left(y_{i}=1\right)}{1-prob\left(y_{i}=1\right)}\right]=\alpha_{i}+\beta_{i}X_{i}+\varepsilon_{i}$$

where $y_{i}$(i=1,2,3,4) denotes a binary variable for adoption decisions of labeled salt information, salt-restriction spoons, reduced condiment use in home cooking and reduced pickled food intake, respectively.$\;y_{i}$ = 1 if the respondent adopts the practice in question, and $y_{i}$= 0 otherwise. *prob* indicates the probability of the individual’s adoption decision.$\;X_{i}\left(i=1,2,\ldots ..,n\right)$ represents a vector of observed explanatory variables. $\beta_{i}$ is a vector of parameters to be estimated and $\alpha_{i}$ is the intercept. $\varepsilon_{i}\left(i=1,2,\ldots ..,n\right)$ is an error term.

#### 2.1.2. Stepwise Logistic Regression

Stepwise logistic regressions based on the Akaike information criterion (AIC) were performed next.

#### 2.1.3. Lasso Logistic Regression

The lasso (least absolute shrinkage and selection operator) is a widely used shrinkage and selection method for linear and nonlinear regressions [33]. Lasso logistic adds a penalty term to Equation (2). The estimation of the vector $\beta_{i}$ is obtained by minimizing the negative log-likelihood function:

(2) $$\;{\hat{\beta }}_{i}=\;\mathrm{argmin}\;\left\{y_{i}-\ln\;\left[1+\exp\left(y_{i}\right)\right]+\;\lambda \left|\beta_{i}\right|\right\}$$

where λ$\left|\beta_{i}\right|$ is the penalty term that penalizes the estimates. The penalty term depends on the positive tuning parameter λ, which controls the tradeoff between the data fitted to the model and the effect of the penalty.

#### 2.1.4. Adaptive Lasso Logistic Model

Adaptive lasso logistic adds $\frac{1}{{\left|{\hat{\beta }}_{i}\right|}^{\gamma }}$ (γ > 0) to different coefficients of $\left|\beta_{i}\right|$ in parameter estimation. The estimation of the vector $\overset{´}{\beta_{i}}$ is as follows:

(3) $$\overset{´}{\beta_{ij}}=\;\mathrm{argmin}\;\left\{y_{i}-\ln\;\left[1+\exp\left(y_{i}\right)\right]+\;\lambda \;\frac{1}{{\left|{\hat{\beta }}_{i}\right|}^{\gamma }}\left|\beta_{i}\right|\right\}$$

In this article, the parameter estimation in the adaptive lasso logistic model is done by means of least angle regression (LAR). It is noted that the adaptive lasso logistic regression model has more advantages than the other three models mentioned above in terms of parameter evaluation. This could be due to the fact that multicollinearity can lead to bias in the estimation of logistic models [34], while random errors and uncertainties are ignored during stepwise estimations in the locally optimal solutions [35]. Moreover, the usual defects of lasso logistic regression—inconsistency and asymptotic non-normality of parameter estimation [36]—are overcome by adaptive lasso logistic regression [37].

The R software package for Windows, version 3.6 (this free package can be downloaded from http://www.r-project.org, Revolution Analytic, Mountain View, California, United States of America), was used for the regression analysis and lasso logistic regression was implemented using the R package glmnet. Prior to regression analysis, the Pearson correlation test was used to test the linearity between potential independent variables and then a Chi-square test was used to assess whether the difference was statistically significant between respondent characteristics and each salt reduction practice. All characteristics whose *p*-value of Chi-square statistic were statistically significant (*p*-value < 0.05) were included as independent variables in subsequent regression models. The models mentioned above were then compared to each other by means of the Akaike information criterion (AIC) and Bayesian information criterion (BIC). The best performing model was identified by the lowest AIC and BIC.

### 2.2. Data

This study developed a survey instrument containing 57 questions (see Supplementary Materials A for details). The cross-sectional survey was conducted in China from 25 June to 15 July 2019 using stratified sampling. Participants aged over 15 years old were recruited to complete an online survey from the China Cloud Panel of GMO E-Lab Marketing Research (Shanghai) Co, LTD. GMO is one of the leading global companies specializing in online data-intensive services (website: https://www.gmo-e-lab.com/cn/index.aspx). In order to collect nationally representative samples, the study employed a proportionate stratified sampling approach to select 230 individuals who were proportionally distributed across age groups according to the age distribution among China’s population—17% for between 15 and 18 years, 24% for between 19 and 30 years, 23% for between 31 and 40 years, 20% for between 41 and 59 years and 16% for 60 years or above—from each of China’s seven geographical regions: East China, South China, North China, Central China, Southwest China, Northwest China and Northeast China. This generated 1610 valid samples (i.e., 230 samples × 7 regions) used for analysis.

## 3. Results

Table A1 from Appendix A presents the respondents’ characteristics. Of the 1610 respondents, 26.40%, 22.98%, 33.54% and 37.20% reported the adoption of labeled salt information, salt-restriction spoons, reduced condiment use in home cooking and reduced pickled food intake, respectively. Moreover, 66.02% of respondents’ education level was junior college or undergraduate, with 48.14% being male and 26.58% at risk of noncommunicable diseases.

It was found that there was no high correlation (˂0.70) between 21 potential independent variables. Moreover, the independent variables with a *p*-value < 0.05 were identified as statistically significant determinants of the relevant salt reduction practice (see Supplementary Materials B for details).

The results are shown in Table A2, Table A3, Table A4 and Table A5 from Appendix A, where the lasso method does not present any *p*-value (see Supplementary Materials C for the estimation details of lasso logistic regression and adaptive lasso logistic regression). The adaptive lasso logistic regression models were identified as the best performing models as they demonstrated the lowest AIC and BIC.

Table A2 from Appendix A presents estimation results for the adoption of labelled salt information. Salt knowledge level, perceived benefits of salt restriction, participation in nutrition education and training programs on sodium reduction, whose β coefficients were 0.023, 0.272, 0.030, were identified as determining variables by adaptive lasso logistic regression, suggesting a positive association between the adoption of labelled salt information and these factors. Respondents who had a higher level of salt knowledge were more likely to read the sodium content contained in food labels. People with perceived benefits from salt restriction tended to believe that reducing salt intake can improve health [38]. Their health awareness motivated them to check labelled salt information in order to choose foods containing a lower salt level. Moreover, nutrition and health training and courses can raise people’s awareness of healthy diets and willingness to make informed choices of food products so as to reduce salt intake. Hence, people who attend the training and courses are more likely to seek salt information from food labels.

Table A3 from Appendix A reports the estimation results for the adoption of salt-restriction spoons. The adaptive lasso logistic model identified that the usage of salt-restriction spoons is positively correlated with having relatives and friends with noncommunicable diseases, hypertension awareness level, knowledge level of healthy diet and having a better dietary habit, whose β coefficients were 0.064, 0.071, 0.113, 0.029, whereas it was negatively correlated with preference for salty foods; most of these results are in line with previous findings in Chen et al. [12]. For example, participants who understood how a healthy diet can contribute to maintaining good health or improving poor health were more motivated to use a salt-restriction spoon.

Table A4 from Appendix A shows the estimation results for reducing condiment usage in home cooking. Age and hypertension awareness level, whose *β* coefficients were 0.042 and 0.170, were found to play positive roles in reducing condiment usage. Conversely, preference for salty food, whose *β* coefficient was −0.098, resulted in greater use of condiments during cooking, mainly because such individuals were more accustomed to a salty taste and dishes tasted bland to them with fewer condiments [13].

Table A5 from Appendix A presents the results for reducing pickled food intake. This study confirmed earlier findings that older respondents and those with higher education levels have a higher propensity to reduce pickled food intake [14,39]. Increased awareness of hypertension also appeared to relate to reduced pickled food intake, as respondents who were aware of their high blood pressure condition may have been informed that pickled food could be detrimental to health. As expected, salt taste preference, whose β coefficient was −0.041, increased people’s pickled food intake.

Finally, all variables identified by the adaptive lasso logistic models were used as independent variables in the logistic regressions to predict the adoption of each salt reduction practice as a robustness check. As shown in Table A6, all independent variables were statistically significant and the size of the estimated coefficients underwent minimal changes compared to those from the adaptive lasso logistic models. This suggested that the results were robust to the different regression models used.

These results reveal several interesting patterns regarding Chinese residents’ adoption of the main salt reduction measures. The positive influence of hypertension awareness and the negative impact of preference for salty foods were found to be statistically significant for most measures except for using salt information on food labels. However, some variables that significantly explained salt reduction practices in previous studies were not found by our analysis, which were respondents’ demographic characteristics, such as gender and health status. The study suggested that factors such as knowledge, perceived benefits, awareness and belief regarding salt intake were more likely than demographic characteristics to be the main determinants of adopting salt reduction measures. This finding suggests that salt reduction strategies need to pay more attention to raising awareness and improving knowledge of the relationship between salt intake and health among the public [40].

## 4. Discussion

The strength of our study was its exploration of most explanatory factors that affect residents’ adoption of salt reduction practices by adopting a series of logistic regression models. However, there are some clear limitations to our study which we will address in future research. For example, the absence of a clear association between salt reduction practices and actual salt consumption suggests that focusing on practices alone may be of limited efficacy [41]. It would be helpful to provide a detailed comparison of changes in salt consumption through salt reduction practices. Secondly, only correct use of labelled salt information and salt-restriction spoons can result in actual salt intake reduction [10,12]. Thus, whether Chinese residents use labelled salt information and/or salt-restriction spoons correctly or not, which was not discussed in our study, warrants further investigation. Finally, it was difficult to quantitatively define “reduced” condiment or pickled food intake due to lack of actual measuring scales in the online survey environment. These may lead to subjective answers and subsequently increase the margin of error. Future research should design easy-to-understand measuring instruments to obtain salt consumption quantities from respondents.

The regression results suggest that few socioeconomic characteristics (e.g., age and education) factored into salt reduction practices but high salt knowledge level and perceived benefits of salt restriction could largely explain the use of labelled salt information. Specifically speaking, salt information on food labels in China is usually presented in the format of numbers and technical terminology (e.g., Nutrient Reference Values) and therefore is not easily understood by people with limited numerical skills and background knowledge [10]. Furthermore, a reasonable level of salt knowledge is needed to convert sodium content to salt levels [16]. Thus, it would be useful to provide specific public education aiming at reducing salt intake in China by improving the knowledge of salt and raising awareness of low-sodium-related benefits. In addition, the remaining three salt reduction practices were largely explained by having awareness of hypertension and taste preference for salty foods. This highlights the need to further increase hypertension awareness in China. It is important to enhance the public’s awareness of hypertension and its relation to salt intake through integrating the hypertension–salt nexus into the existing noncommunicable disease education programs. Moreover, awareness regarding a low-salt diet has been found to affect the use of salt information on food labels [38]. It may be difficult in the short term to adjust the taste preference for salty food among Chinese people, particularly those from Northern China [40]. However, interventions are needed to change taste choices towards reduced salt at the population level. A specific focus could be children’s diets, as preferential changes among these cohorts can have long-lasting effects on food healthfulness in future decades.

## 5. Conclusions

This study attempted to explore factors associated with Chinese residents’ adoption of four salt reduction measures: labelled salt information, salt-restriction spoons, reduced condiment usage in home cooking and reduced pickled food intake. Based on a large-scale national representative online survey (N = 1610), this study employed multiple regression modelling techniques, including an adaptive lasso logistic regression, to identify the key determinants of adoption. The findings suggest that the majority of respondents did not adopt these measures, resulting in fairly low adoption levels. (1) Adoption of labelled salt information was positively correlated with salt knowledge level, perceived benefits of salt restriction, participation in nutrition education and training programs on sodium reduction. (2) Adoption of the salt-restriction spoon had positive linkages with relatives and friends with noncommunicable diseases, having awareness of hypertension, knowing about healthy diets and having better dietary habits, whereas taste preference for salty foods was a barrier to implementation. (3) Adoption of reduced condiment usage in home cooking was positively correlated with age and awareness of hypertension, but taste preference for salty foods played a negative role. (4) Adoption of reduced pickled food intake had a negative correlation with age, education and having awareness of hypertension, but taste preference for salty foods posed a negative influence.

## Data Availability

Data is contained within the article or supplementary material.

## Supplementary Materials

The following are available online at https://www.mdpi.com/1660-4601/18/2/445/s1, Supplementary Materials A: The questionnaire; Supplementary Materials B: Chi-square test results of subgroups from adoption of salt reduction practices; Supplementary Materials C: Estimation details; Supplementary Materials D: The online salt knowledge questionnaire.

## Appendix A

**Table A1:** Variables Description and Summary Statistics.

Variables	Definition	Samples	Percentage %
Labeled salt information usage	0 = No	1185	73.60
	1 = Yes	425	26.40
Salt-restriction spoon usage	0 = No	1240	77.02
	1 = Yes	370	22.98
Less condiments added in home cooking	0 = No	1070	66.46
	1 = Yes	540	33.54
Reduced pickled food intake	0 = No	1011	62.80
	1 = Yes	599	37.20
Gender	0 = Female	835	51.86
	1 = Male	775	48.14
Age	1 = from 15 to 18 years old	278	17.27
	2 = from 19 to 30 years old	381	23.66
	3 = from 31 to 40 years old	364	22.61
	4 = from 41 to 59 years old	323	20.06
	5 = 60 years old or above	264	16.40
Education	1 = Primary school or below	19	1.18
	2 = Junior school	101	6.27
	3 = Senior school or above	341	21.18
	4 = Junior college or undergraduate	1063	66.02
	5 = Postgraduate or above	86	5.34
Employment	0 = No	656	40.75
	1 = Yes	954	59.25
Individual annual income (Yuan)	1 ≤ 10,000	369	22.92
	2 = 10,001–20,000	66	4.10
	3 = 20,001–30,000	73	4.53
	4 = 30,001–40,000	111	6.89
	5 = 40,001–50,000	164	10.19
	6 = 50,001–80,000	296	18.39
	7 = 80,001–100,000	187	11.61
	8 = 100,001–150,000	171	10.62
	9 = 150,001–200,000	91	5.65
	10 = 200,001–300,000	47	2.92
	11 = 300,001–500,000	16	0.99
	12 = 500,001–1,000,000	13	0.81
	13 = 1,000,001–2,000,000	5	0.31
	14 ≥ 2,000,000	1	0.06
Live in urban areas	0 = No	411	25.53
	1 = Yes	1199	74.47
Self-rated physical health	1 = Very poor	6	0.37
	2 = Poor	58	3.60
	3 = general	619	38.45
	4 = Good	666	41.37
	5 = Very good	261	16.21
Overweight/obesity ^a^	0 = No	1119	69.50
	1 = Yes	491	30.50
Noncommunicable diseases	0 = No	1182	73.42
	1 = Yes	428	26.58
Relatives and friends with noncommunicable diseases	0 = No	1143	70.99
	1 = Yes	467	29.01
Salt knowledge level ^b^	1 = Low	303	18.82
	2 = Medium	581	36.09
	3 = High	610	37.89
	4 = Very high	116	7.20
Having awareness of hypertension	0 = No	113	7.02
	1 = Yes	1497	92.98
Knowledge about healthy diets ^c^	0 = No	1108	68.82
	1 = Yes	502	31.18
Positive attitudes towards a low-salt diet	0 = No	560	34.78
	1 = Yes	1050	65.22
Concerned about salt in diet	0 = No	340	21.12
	1 = Yes	1270	78.88
Perceive benefits of salt restriction	0 = No	1070	66.46
	1 = Yes	540	33.54
Taste preference for salty foods	0 = No	1336	82.98
	1 = Yes	274	17.02
Dietary habit ^d^	1 = Very unhealthy	50	3.11
	2 = unhealthy	473	29.38
	3 = general	506	31.43
	4 = healthy	326	20.25
	5 = Very healthy	255	15.84
Participation in nutrition education and training programs on sodium reduction	0 = No/Unknown	1222	75.90
	1 = Yes	388	24.10
Community-based education on salt reduction availability	0 = No/Unknown	1158	71.93
	1 = Yes	452	28.07
Salt intake increase by food advertising	0 = No/Unknown	1591	98.82
	1 = Yes	19	1.18

^a^ A respondent is defined as overweight/obese if his/her BMI is ≥24. ^b^ Each respondent’s salt knowledge level was evaluated by the twenty declarative knowledge questions that were included in the online questionnaire (see Supplementary Materials D for details). The correct answer proportions of 0–25%, 26–50%, 51–75% and 76–100% indicate low level, medium level, high level and very high level, respectively. ^c^ Whether respondents know about healthy diets or not was evaluated by five questions from *The Chinese Dietary Guidelines* [9]. These were included in the online questionnaire (see Supplementary Materials A for details). A respondent would be defined as knowing about healthy diets if all the questions were answered as “Yes”, and “No” otherwise. ^d^ Each respondent’s level was evaluated by fourteen questions designed by *The Chinese Dietary Guidelines* [9]. These were included in the online questionnaire (see Supplementary Materials A for details). The proportions of “Yes” answers of 0–20%, 21–40%, 41–60%, 61–80% and 81–100% indicate very unhealthy, unhealthy, general, healthy and very healthy, respectively.

**Table A2:** Results for Adoption of Labelled Salt Information.

The Various Forms of Logistic Regression	Logistic Regression			Stepwise Logistic Regression			Lasso Logistic Regression	Adaptive Lasso Logistic Regression
Variables	*Β* (SE)	*OR* (95%CI)	*p*-value	*β* (SE)	*OR* (95%CI)	*p*-value	*β*	*β*
Age	0.072 (0.056)	1.074 (0.962–1.199)	0.203	0.097 (0.048)	1.102 (1.004–1.210)	0.041	—	—
Education	0.039 (0.100)	1.040 (0.854–1.265)	0.699	—	—	—	—	—
Employment	0.200 (0.165)	1.222 (0.884–1.687)	0.225	0.313 (0.127)	1.368 (1.066–1.755)	0.014	—	—
Individual annual income	0.023 (0.030)	1.024 (0.965–1.086)	0.441	—	—	—	0.008	—
Live in the urban areas	0.077 (0.165)	1.081 (0.782–1.493)	0.639	—	—	—	—	—
Salt knowledge level	0.172 (0.079)	1.188 (1.017–1.388)	0.030	0.209 (0.075)	1.233 (1.064–1.428)	0.005	0.029	0.023
Having awareness of hypertension	0.708 (0.337)	2.029 (1.049–3.926)	0.036	0.760 (0.334)	2.138 (1.110–4.118)	0.023	—	—
Positive attitudes towards a low-salt diet	0.051 (0.136)	1.052 (0.806–1.373)	0.708	—	—	—	—	—
Perceived benefits ofsalt restriction	1.312 (0.122)	3.713 (2.923–4.717)	0.000	1.315 (0.122)	3.726 (2.935–4.732)	0.000	0.970	0.272
Participation in nutrition education and training program on sodium reduction	0.305 (0.148)	1.356 (1.015–1.813)	0.039	0.356 (0.142)	1.427 (1.081–1.884)	0.012	—	0.030
Community-based education on salt reduction availability	0.136 (0.144)	1.146 (0.864–1.521)	0.344	—	—	—	—	—
Constant	−3.461 (0.499)	0.031 (0.012–0.083)	0.000	−3.360 (0.389)	0.035 (0.016–0.074)	0.000	−1.510	0.112
Observation	1610			1610			1610	1610
AIC	1695.1			1687.78			1668.4	1587.5
BIC	1759.7			1725.47			1733.0	1652.1
Optimal λ	—			—			0.040	—

**Table A3:** Estimation Results for Adoption of Salt-Restriction Spoons.

The Various Forms of Logistic Regression	Logistic Regression			Stepwise Logistic Regression			Lasso Logistic Regression	Adaptive Lasso Logistic Regression
Variables	*β* (SE)	*OR* (95%CI)	*p*-value	*β* (SE)	*OR* (95%CI)	*p*-value	*β*	*β*
Age	0.040 (0.053)	1.041 (0.938–1.155)	0.453	—	—	—	—	—
Live in the urban areas	0.089(0.164)	1.093 (0.793–1.508)	0.586	—	—	—	—	—
Relatives and friends with noncommunicable diseases	0.416 (0.132)	1.516 (1.171–1.963)	0.002	0.450 (0.130)	1.569 (1.217–2.022)	0.001	—	0.064
Salt knowledge level	0.097 (0.081)	1.102 (0.940–1.291)	0.233	0.126 (0.074)	1.134 (0.980–1.311)	0.091	—	—
Having awareness of hypertension	0.942 (0.360)	2.567 (1.268–5.196)	0.009	0.946 (0.359)	2.576 (1.274–5.210)	0.008	—	0.071
Know about healthy diet	0.520 (0.139)	1.682 (1.281–2.207)	0.000	0.572 (0.135)	1.772 (1.360–2.308)	0.000	0.332	0.113
Taste preference for salty foods	−0.484 (0.180)	0.616 (0.433–0.877)	0.007	−0.480 (0.180)	0.619 (0.435–0.880)	0.008	—	−0.046
Dietary habit	0.181 (0.059)	1.199 (1.069–1.344)	0.002	0.194 (0.058)	1.214 (1.084–1.360)	0.001	0.056	0.029
Participation in nutrition education and training program on sodium reduction	0.116 (0.150)	1.123 (0.837–1.508)	0.439	—	—	—	—	—
Community-based education on salt reduction availability	0.114 (0.145)	1.120 (0.842–1.490)	0.435	—	—	—	—	—
Constant	−3.430 (0.435)	0.032 (0.014–0.076)	0.000	−3.320 (0.406)	0.036 (0.016–0.080)	0.000	−1.500	0.026
Observation	1610			1610			1610	1610
AIC	1658.5			1653.42			1640.2	1593.5
BIC	1717.7			1691.11			1699.4	1655.3
Optimal λ	—			—			0.043	—

**Table A4:** Estimation Results for Adoption of Reduced Condiment Usage in Home Cooking.

The Various Forms of Logistic Regression	Logistic Regression			Stepwise Logistic Regression			Lasso Logistic Regression	Adaptive Lasso Logistic Regression
Variables	*β* (SE)	*OR* (95%CI)	*p*-value	*β* (SE)	*OR* (95%CI)	*p*-value	*β*	*β*
Age	0.206 (0.050)	1.229 (1.114–1.355)	0.000	0.228 (0.042)	1.256 (1.157–1.365)	0.000	0.103	0.042
Education	0.134 (0.089)	1.143(0.960–1.361)	0.132	0.190 (0.079)	1.210 (1.036–1.412)	0.016	—	—
Employment	0.109 (0.146)	1.115 (0.837–1.485)	0.457	—	—	—	—	—
Individual annual income	0.014 (0.027)	1.014 (0.961–1.069)	0.611	—	—	—	0.016	—
Live in the urban areas	0.042 (0.147)	1.043 (0.782–1.391)	0.775	—	—	—	—	—
Relatives and friends with noncommunicable diseases	0.188 (0.120)	1.207 (0.954–1.527)	0.118	0.211 (0.119)	1.234 (0.978–1.559)	0.077	—	—
Having awareness of hypertension	1.155 (0.289)	3.173 (1.801–5.589)	0.000	1.179 (0.288)	3.250 (1.847–5.720)	0.000	0.450	0.170
Taste preference for salty foods	−0.657 (0.158)	0.519 (0.381–0.706)	0.000	−0.648 (0.158)	0.523 (0.384–0.712)	0.000	−0.214	−0.098
Salt intake incrase by food advertising	−0.893 (0.645)	0.409 (0.116–1.449)	0.166	−0.882 (0.646)	0.414 (0.117–1.467)	0.172	—	—
Constant	−3.010 (0.427)	0.049 (0.021–0.114)	0.000	−3.147 (0.415)	0.043 (0.019–0.097)	0.000	−1.458	0.069
Observation	1610			1610			1610	1610
AIC	1985.2			1981.11			1660.4	1590.9
BIC	2049.4			2018.79			1724.6	1644.7
Optimal λ	—			—			0.031	—

**Table A5:** Estimation Results for Adoption of Reduced Pickled Food Intake.

The Various Forms of Logistic Regression	Logistic Regression			Stepwise Logistic Regression			Lasso Logistic Regression	Adaptive Lasso Logistic Regression
Variables	*β* (SE)	*OR* (95%CI)	*p*-value	*β* (SE)	*OR* (95%CI)	*p*-value	*β*	*β*
Age	0.193 (0.048)	1.213 (1.103–1.333)	0.000	0.223 (0.041)	1.250 (1.154–1.353)	0.000	0.106	0.043
Education	0.194 (0.087)	1.215 (1.025–1.440)	0.025	0.240 (0.077)	1.271 (1.092–1.479)	0.002	0.085	0.041
Employment	0.067 (0.142)	1.069 (0.810–1.412)	0.637	—	—	—	—	—
Individual annual income	0.008 (0.026)	1.008 (0.958–1.061)	0.757	—	—	—	0.109	—
Live in the urban areas	0.138 (0.142)	1.148 (0.870–1.516)	0.329	—	—	—	0.007	—
Having awareness of hypertension	0.615 (0.240)	1.850 (1.155–2.963)	0.011	0.633 (0.240)	1.883 (1.177–3.012)	0.008	0.194	0.093
Positive attitudes towards a low-salt diet	0.167 (0.114)	1.182 (0.945–1.479)	0.144	0.179 (0.114)	1.196 (0.956–1.495)	0.117	—	—
Taste preference for salty foods	−0.346 (0.145)	0.707 (0.533–0.939)	0.017	−0.335 (0.144)	0.715 (0.539–0.950)	0.020	−0.016	−0.041
Constant	−2.639 (0.387)	0.071 (0.033–0.152)	0.000	−2.736 (0.373)	0.065 (0.031–0.135)	0.000	−1.393	0.014
Observation	1610			1610			1610	1610
AIC	2081.3			2077.17			1630.4	1596.8
BIC	2129.8			2109.47			1699.0	1645.2
Optimal λ	—			—			0.027	—

**Table A6:** Robustness Checks on the Estimation from Adaptive Lasso Logistic Model.

**Salt Reduction Practices**	Labeled Salt Information Usage			Salt-Restriction Spoon Usage			Reduced Condiment Usage in Home Cooking			Reduced Pickled Food Intake		
	*β* (SE)	*OR* (95%CI)	*p*-value	*β* (SE)	*OR* (95%CI)	*p*-value	*β* (SE)	*OR* (95%CI)	*p*-value	*β* (SE)	*OR* (95%CI)	*p*-Value
Age	—		—	—		—	0.233 (0.041)	1.262 (1.164–1367)	0.000	0.218 (0.040)	1.243 (1.149–1.345)	0.000
Education	—		—	—		—	—		—	0.257 (0.076)	1.293 (1.113–1.502)	0.001
Relatives and friends with noncommunicable diseases	—		—	0.464 (0.129)	1.591 (1.235–2.050)	0.000	—		—	—		—
Salt knowledge level	0.222 (0.074)	1.249 (1.081–1.444)	0.003	—		—	—		—	—		—
Having awareness of hypertension	—		—	1.012 (0.357)	2.752 (1.366–5.543)	0.005	1.277 (0.285)	3.588 (2.051–6.276)	0.000	0.671 (0.239)	1.956 (1.225–3.121)	0.005
Know about healthy diet	—		—	0.621 (0.132)	1.862 (1.438–2.410)	0.000	—		—	—		—
Perceived benefits of salt restriction	1.397 (0.120)	4.043 (3.196–5.116)	0.000	—		—	—		—	—		—
Taste preference for salty foods	—		—	−0.487 (0.180)	0.614 (0.432–0.874)	0.007	−0.637 (0.157)	0.529 (0.389–0.719)	0.000	−0.336 (0.144)	0.715 (0.032–0.139)	0.020
Dietary habit	—		—	0.193 (0.058)	1.213 (1.083–1.358)	0.000	—		—	—		—
Participation in nutrition education and training program on sodium reduction	0.371 (0.141)	1.449 (1.099–1.910)	0.009	—		—	—		—	—		—
