# Supplementary material for: Factors Affecting Salt Reduction Measure Adoption among Chinese Residents
Source: Int J Environ Res Public Health. 2021 Jan 8;18(2):445. doi: 10.3390/ijerph18020445 (PMC7827444; doi:10.3390/ijerph18020445)
Supplement: Supplementary file 1 [file ijerph-18-00445-s001.pdf]

International Journal of Environmental Research and Public Health

MDPI

Supplementary Materials A: The Questionnaire

Section 1 Adoption of Main Salt Reduction Measures

As we know, reducing excessive salt intake is important to help keep us healthy.
So
Q1-1 Do you generally read labeled salt information (See Figure1 for detail) before making purchase decision on pre-package food? A. Yes B. No
Q1-2 Do you generally use salt-restriction spoon (See Figure2 for detail) to calculate the amount of salt? A. Yes B. No
Q1-3 Do you generally add less condiment (mainly soy source, fish source) during home cooking?
A. Yes B. No
Q1-4 Do you generally have less pickled food intake(*e.g.*, pickled vegetables, pickled meat, pickled seafood)? A. Yes B. No

营养成分表

项目	每100g	NRV%
能量	2505kJ	30%
蛋白质	27.0g	45%
脂 肪	50.2g	84%
碳水化合物	16.5g	6%
钠	618mg	31%

Figure S1. labeled salt information indicated in the last row of nutritional information panel.

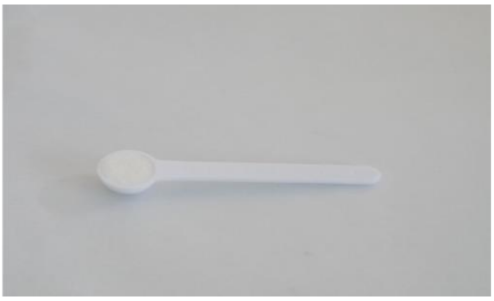

Figure S2. the salt-restriction spoon.

Section 2 Demographic Characteristics

Q2-1 Your gender : A. Male B. Female
Q2-2 Your age: A.15–18 years old B. 19–30 years old C.31–40 years old
D.41-59 years old E. at least 60 years old

Q2-3 Your education level: A. Primary school or below B. Junior school C. Senior school D. Junior college or undergraduate E. Postgraduate or above

Q2-4 Are you now being employed: A. Yes B. NoQ2-5 Your individual annual income on average (Yuan):

A.≤10,000 B.10,001–20,000 C.20,001–30,000 D.30,001–40,000 E.40,001–50,000 F.50,001–80,000 G.80,001–100,000 H.100,001–150,000 I.150,001–200,000 J.200,001–300,000 K.300,001–500,000 L.500,001–1,000,000 M.1,000,001–2,000,000; N.≥2,000,000

Q2-6 Where do you live at present? A. The rural area B. The urban area

Q2-7 In general, how would you rate your health status:

A.Very poor B.Poor C.Fair D. Good E. Very good

Q2-8 **measurement of whether respondents are overweight (24.0≤BMI < 28.0 kg/m^2) and obesity(BMI≥28.0 kg/m^2):** Your height: ______cm; Your weight:______kg.

Q2-9 Have you had noncommunicable diseases (*e.g.,* hypertension, cardiovascular disease and stroke)? A. Yes B. No

Q2-10 Have your relatives and friends had noncommunicable diseases?

A. Yes B. No

Section 3 Salt Knowledge

Q3 **measurement of your salt knowledge level:**

Q3-1 What do you know the China dietary recommendations below?

What is the relationship between salt and sodium?

How many grams of salt is equivalent to one standard teaspoon of salt?

How many sodium per 100 g or 100 ml contain in a pre-packaged food when considered as 'low in salt'?

What is the maximum recommended daily amount of salt for an adult in China?

Q3-2Which conditions below might be associated with high salt intakes? (multiple choices)

A. High blood pressure B. High blood sugar C. Stroke D. Kidney disease E. Osteoporosis

Q3-3 What is salt content of commonly eaten foods below?

White bread: A.Low B.Medium C.High

Bacon: A.Low B.Medium C.High

Boiled white rice: A.Low B.Medium C.High

Uncooked beef steak: A.Low B.Medium C.High

Frozen mix vegetables: A.Low B.Medium C.High

Corn flakes: A.Low B.Medium C.High

Fresh food: A.Low B.Medium C.High

Fast foods: A.Low B.Medium C.High

Q3-4 Which statements below are misconceptions?(multiple choices)

A.Sea salt is better than table salt

B,Drinking more water can neutralise salt in my diet

C.Cutting down on salt causes leg cramps

Section 4 Dietary Habit

Q4 **measurement of health level of your dietary habit:**

Q4-1 Do you have 12 kinds of food every day? A. Yes B. No

Q4-2 Do you have staple food (*e.g.*,cereals) at each meal? A. Yes B. No

Q4-3 Do you have vegetables at each meal? A. Yes B. No

Q4-4 Do you have fruit every day? A. Yes B. No

Q4-5 Do you have milk or other dairy products every day? A. Yes B. No

Q4-6 Do you have beans or soy products every day? A. Yes B. No

Q4-7 Do you have an adequate amount of fish every week? A. Yes B. No

Q4-8 Do you have an adequate amount of livestock and poultry every week?
A. Yes B. No

Q4-9 Do you have 4-7 eggs every week? A. Yes B. No

Q4-10 Do you have 1500-1700 ml of water every day? A. Yes B. No

Q4-11Do you rarely drink alcohol? A. Yes B. No

Q4-12 Do you rarely have sugary drinks? A. Yes B. No

Q4-13 Do you rarely have salty food? A. Yes B. No

Q4-14 Do you rarely have fried food? A. Yes B. No

Section 5 Healthy Diet

Q5 **measurement of whether participants know about healthy diet**

Q5-1 Chinese people should stick to the principle of "food diversity, mainly cereals". A. Yes B. No

Q5-2 Chinese people should stick to the principle of "balanced eating and healthy weight". A. Yes B. No

Q5-3 Chinese people should stick to eating more vegetables, milk and soy. A. Yes B. No

Q5-4 Chinese people should stick to eating fish, poultry, eggs and lean meat in moderation.A. Yes B. No

Q5-5 Chinese people should stick to the principle of "less salt, less oil, control sugar and limit alcohol". A. Yes B. No

Section 6 Attitude/Belief and Behaviors Related to Salt Intake

Q6-1 Do you have awareness of hypertension? A. Yes B. No

Q6-2 What is your attitudes towards a low-salt diet? A. Positive B.Negative

Q6-3 Do you concern about salt in diet? A. Yes B. No

Q6-4 Do you know salt-restriction is beneficial? A. Yes B. No

Q6-5 Do you have beliefs about the taste of salt? A. Yes B. No

Q6-6 Have you ever participated in nutrition education and training program on sodium reduction? A. Yes B. No C. Don't know

Q6-7 Are there any education program on salt reduction in the community?
A. Yes B. No C. Don't know

Q6-8 Does the food advertising increase your salt intake? A. Yes B. No C. Don't know

Supplementary Materials B: Chi-Square Test Results of Subgroups from Adoption of Salt Reduction Practices

Table S1. Chi-Square Test Results of Subgroups from Adoption of Labeled Salt Information.

Subgroups	**Labeled Salt Information Usage = 1[[*n*(%)]**	**Labeled Salt Information Usage = 0[*n* (%)]**	**Chi-Square**	***p*-value**
Male				
Male = 0(*n* = 835)	227(27.19)	608(72.81)	0.554	0.456
Male = 1(*n* = 775)	198(25.55)	577(74.45)		
Age				
Age = 1(*n* = 278)	47(16.91)	231(83.09)		
Age = 2(*n* = 381)	96(25.20)	285(74.80)		
Age = 3(*n* = 364)	110(30.22)	254(69.78)	18.461	0.001
Age = 4(*n* = 323)	95(29.41)	228(70.59)		
Age = 5(*n* = 264)	77(29.17)	187(70.83)		
Education				
Education = 1(*n* = 19)	1(5.26)	18(94.74)		
Education = 2(*n* = 101)	19(18.81)	82(81.19)		
Education = 3(*n* = 341)	83(24.34)	258(75.66)	10.651	0.031
Education = 4(*n* = 1063)	294(27.66)	769(72.34)		
Education = 5(*n* = 86)	28(32.56)	58(67.44)		
Employment				
Employment = 0(*n* = 656)	138(21.04)	518(78.96)	16.376	0.000
Employment = 1(*n* = 954)	287(30.08)	667(69.92)		
Individual annual income				
Individual annual income = 1(*n* = 369)	56(15.18)	313(84.82)		
Individual annual income = 2(*n* = 66)	18(27.27)	48(72.73)		
Individual annual income = 3(*n* = 73)	18(24.66)	55(75.34)		
Individual annual income = 4(*n* = 111)	25(22.52)	86(77.48)		
Individual annual income = 5(*n* = 164)	47(28.66)	117(71.34)		
Individual annual income = 6(*n* = 296)	95(32.09)	201(67.91)		
Individual annual income = 7(*n* = 187)	61(32.62)	126(67.38)	40.523	0.000
Individual annual income = 8(*n* = 171)	57(33.33)	114(66.67)		
Individual annual income = 9(*n* = 91)	25(27.47)	66(72.53)		
Individual annual income = 10(*n* = 47)	11(23.40)	36(76.60)		

Individual annual income = 11(n = 16)	3(21.25)	13(78.75)		
Individual annual income = 12(n = 13)	5 (38.46)	8(61.54)		
Individual annual income = 13(n = 5)	2 (40.00)	3 (60.00)		
Individual annual income =14(n = 1)	0(0.00)	1 (100.00)		
Live in urban areas				
Live in urban areas = 0(n = 411)	84(20.44)	327(79.56)	10.088	0.001
Live in urban areas = 1(n = 1199)	341(28.44)	858(71.56)		
Overweight/obesity				
Overweight/obesity= 0(n = 1119)	295 (26.36)	824 (73.64)	0.002	0.962
Overweight/obesity = 1(n = 491)	130（26.48）	361（73.52）		
Relatives and friends with noncommunicable diseases				
Relatives and friends with noncommunicable diseases = 0(n = 1143)	295（25.81）	848（74.19）	0.702	0.402
Relatives and friends with noncommunicable diseases = 1(n = 467)	130（27.84）	337（72.16）		
Salt knowledge level				
Salt knowledge level = 1(n = 303)	52（17.16）	251（82.84）		
Salt knowledge level = 2(n = 581)	147（25.30）	434（74.70）	22.218	0.000
Salt knowledge level = 3(n = 610)	188（30.82）	422（69.18）		
Salt knowledge level = 4(n = 116)	38（32.76）	78（67.24）		
Having awareness of hypertension				
Having awareness of hypertension = 0(n = 113)	11（9.73）	102（90.27）	17.367	0.000
Having awareness of hypertension =1(n = 1497)	414（27.66）	1083（72.34）		
Positive attitudes towards a low-salt diet				

Positive attitudes towards a low-salt diet = 0(n = 560)	129 (23.04)	431 (76.96)	4.995	0.025
Positive attitudes towards a low-salt diet = 1(n = 1050)	296 (28.19)	754 (71.81)		
Concerned about salt in diet				
Concerned about salt in diet = 0(n = 340)	98 (28.82)	242 (71.18)	1.306	0.253
Concerned about salt in diet = 1(n = 1270)	327 (25.75)	943 (74.25)		
Perceived benefits of salt-restriction				
Perceived benefits of salt-restriction = 0(n = 1070)	181 (16.92)	889 (83.08)	147.615	0.000
Perceived benefits of salt-restriction = 1(n = 540)	244 (45.19)	296 (54.81)		
Taste preference for salty foods				
Taste preference for salty foods = 0(n = 1336)	361 (27.02)	975 (72.98)	1.570	0.210
Taste preference for salty foods = 1(n = 274)	64 (23.36)	210 (76.64)		
Participation in nutrition education and training program on sodium reduction				
Participation in nutrition education and training program on sodium reduction = 0(n = 1222)	294 (24.06)	928 (75.94)	14.273	0.000
Participation in nutrition education and training program on sodium reduction = 1(n = 388)	131 (33.76)	257 (66.24)		
Community-based education on salt reduction availability				
Community-based education on salt reduction availability = 0(n = 1158)	278 (24.01)	880 (75.99)	12.133	0.000

Community-based education on salt reduction availability = 1(n = 452)	147（32.52）	305（67.48）		
Salt intake increase by food advertising				
Salt intake increase by food advertising = 0(n = 1591)	422（26.52）	1169(73.48)	1.114	0.291
Salt intake increase by food advertising = 1(n = 19)	3（15.79）	16(84.21)		

Source: Authors' own calculations.

Table 2. Chi-Square Test Results of Subgroups from Adoption of Salt-Restriction Spoon.

Subgroups	**Salt-Restriction Spoon Usage = 1 [n (%)]**	**Salt-Restriction Spoon Usage =0 [n (%)]**	**Chi-Square**	***p*-value**
Male				
Male = 0(n = 835)	203(24.31)	632(75.69)	1.734	0.188
Male = 1(n = 775)	167(21.55)	608(78.45)		
Age				
Age = 1(n = 278)	48(17.27)	230(82.73)		
Age = 2(n = 381)	83(21.78)	298(78.22)		
Age =3(n=364)	81(22.25)	283(77.75)	11.324	0.023
Age =4(n=323)	91(28.17)	232(71.83)		
Age = 5(n = 264)	67(25.38)	197(74.62)		
Education				
Education = 1(n = 19)	1(5.26)	18(94.74)		
Education = 2(n = 101)	23(22.77)	78(77.23)		
Education = 3(n = 341)	72(21.11)	269(78.89)	4.549	0.337
Education = 4(n = 1063)	254(23.89)	809(76.11)		
Education = 5(n = 86)	20(23.26)	66(76.74)		
Employment				
Employment = 0(n = 656)	139(21.19)	517(78.81)	2.009	0.156
Employment = 1(n = 954)	231(24.21)	723(75.79)		
Individual annual income				
Individual annual income = 1(n = 369)	72(19.51)	297(80.49)		
Individual annual income = 2(n = 66)	8(12.12)	58(87.88)		
Individual annual income = 3(n = 73)	17(23.29)	56(76.71)		
Individual annual income = 4(n = 111)	20(18.02)	91(81.98)		
Individual annual income = 5(n = 164)	42(25.61)	122(74.39)		

Individual annual income = 6(n = 296)	79(26.69)	217(73.31)		
Individual annual income = 7(n = 187)	41(21.93)	146(78.07)	21.011	0.073
Individual annual income = 8(n = 171)	53(30.99)	118(69.01)		
Individual annual income = 9(n = 91)	22(24.18)	69(75.82)		
Individual annual income = 10(n = 47)	9(19.15)	38(80.85)		
Individual annual income = 11(n = 16)	5(31.25)	11(68.75)		
Individual annual income = 12(n = 13)	2(15.38)	11(84.62)		
Individual annual income = 13(n = 5)	0(0.00)	5(100.00)		
Individual annual income = 14(n = 1)	0(0.00)	1(100.00)		
Live in urban areas				
Live in urban areas = 0(n = 411)	74(18.00)	337(82.00)	7.722	0.005
Live in urban areas = 1(n = 1199)	296 (24.69)	903(75.31)		
Overweight/obesity				
Overweight/obesity= 0(n = 1119)	259 (23.15)	860 (76.85)	0.056	0.813
Overweight/obesity = 1(n = 491)	111 (22.61)	380 (77.39)		
Noncommunicable diseases				
Noncommunicable diseases = 0(n = 1182)	263 (22.25)	919 (77.75)	1.342	0.247
Noncommunicable diseases = 1(n = 428)	107 (25.00)	321 (75.00)		
Relatives and friends with noncommunicable diseases				
Relatives and friends with noncommunicable diseases = 0(n = 1143)	231 (20.21)	912 (79.79)	17.099	0.000
Relatives and friends with noncommunicable diseases = 1(n = 467)	139 (29.76)	328 (70.24)		
Salt knowledge level				
Salt knowledge level = 1(n = 303)	51 (16.83)	252 (83.17)		
Salt knowledge level = 2(n = 581)	123 (21.17)	458 (78.83)	16.038	0.001

Salt knowledge level = 3(*n* = 610)	159 (26.07)	451 (73.93)		
Salt knowledge level = 4(*n* = 116)	37 (31.90)	79 (68.10)		
Having awareness of hypertension				
Having awareness of hypertension = 0(*n* = 113)	9 (7.96)	104 (92.04)	15.483	0.000
Having awareness of hypertension = 1(*n* = 1497)	361 (24.11)	1136 (75.89)		
Know about healthy diet				
Know about healthy diet = 0(*n* = 1108)	199 (17.96)	909 (82.04)	50.616	0.000
Know about healthy diet = 1(*n* = 502)	171 (34.06)	331 (65.94)		
Positive attitudes towards a low-salt diet				
Positive attitudes towards a low-salt diet = 0(*n* = 560)	117 (20.89)	443 (79.11)	2.116	0.146
Positive attitudes towards a low-salt diet = 1(*n* = 1050)	253 (24.10)	797 (75.90)		
Taste preference for salty foods				
Taste preference for salty foods = 0(*n* = 1336)	325 (24.33)	1011 (75.67)	8.023	0.005
Taste preference for salty foods =1(*n* = 274)	45 (16.42)	229 (83.58)		
Dietary habit				
Dietary habit = 1(*n* = 50)	6 (12.00)	44 (88.00)		
Dietary habit = 2(*n* = 473)	91 (19.24)	382 (80.76)		
Dietary habit = 3(*n* = 506)	91 (17.98)	415 (82.02)	45.015	0.000
Dietary habit = 4(*n* = 326)	88 (26.99)	238 (73.01)		
Dietary habit = 5(*n* = 255)	94 (36.86)	161 (63.14)		
Participation in nutrition education and training program on sodium reduction				

Participation in nutrition education and training program on sodium reduction = 0(n = 1222)	258 (21.11)	964 (78.89)	10.001	0.002
Participation in nutrition education and training program on sodium reduction = 1(n = 388)	112 (28.87)	276 (71.13)		
Community-based education on salt reduction availability				
Community-based education on salt reduction availability = 0(n = 1158)	239 (20.64)	919 (79.36)	12.786	0.000
Community-based education on salt reduction availability =1(n = 452)	131 (28.98)	321 (71.02)		
Salt intake increase by food advertising				
Salt intake increase by food advertising = 0(n = 1591)	368 (23.13)	1223 (76.87)	1.685	0.194
Salt intake increase by food advertising = 1(n = 19)	2 (10.53)	17 (89.47)		

Source: Author's own calculations

Table 3. Chi-Square Test Results of Subgroups from Adoption of Less Condiment Added in Home Cooking.

Subgroups	**Less Condiment Added in Home Cooking =1[n (%)]**	**Less Condiment Added in Home Cooking =0[n (%)]**	**Chi-Square**	***p*-value**
Male				
Male = 0 (n = 835)	281 (33.65)	554 (66.35)	0.010	0.921
Male = 1(n = 775)	259 (33.42)	516 (66.58)		
Age				
Age = 1(n = 278)	52 (18.71)	226 (81.29)		
Age = 2(n = 381)	125 (32.81)	256 (67.19)		
Age = 3(n = 364)	125 (34.34)	239 (65.66)	41.692	0.000
Age = 4(n = 323)	137 (42.41)	186 (57.59)		
Age = 5(n = 264)	101 (38.26)	163 (61.74)		
Education				
Education = 1(n = 19)	4 (21.05)	15 (78.95)		
Education = 2(n = 101)	32 (31.68)	69 (68.32)		
Education = 3(n = 341)	87 (25.51)	254 (74.49)	41.692	0.004

Education = 4(n = 1063)	384（36.12）	679（63.88）		
Education = 5(n = 86)	33（38.37）	53（61.63）		
Employment				
Employment = 0(n = 656)	188（28.66）	468（71.34）	11.837	0.001
Employment = 1(n = 954)	352（36.90）	602（63.10）		
Individual annual income				
Individual annual income = 1(n = 369)	87（23.58）	282（76.42）		
Individual annual income = 2(n = 66)	19（28.79）	47（71.21）		
Individual annual income = 3(n = 73)	27（36.99）	46（63.01）		
Individual annual income = 4(n = 111)	30（27.03）	81（72.97）		
Individual annual income = 5(n = 164)	53（32.32）	111（67.68）		
Individual annual income = 6 (n = 296)	122（41.22）	174（58.78）	34.758	0.001
Individual annual income = 7(n = 187)	72（38.50）	115（61.50）		
Individual annual income = 8(n = 171)	62（36.26）	109（63.74）		
Individual annual income = 9(n = 91)	36（39.56）	55（60.44）		
Individual annual income = 10(n = 47)	17（36.17）	30（63.83）		
Individual annual income = 11(n = 16)	7（43.75）	9（56.25）		
Individual annual income = 12(n = 13)	5（38.46）	8（61.54）		
Individual annual income = 13(n = 5)	3（60.00）	2（40.00）		
Individual annual income = 14(n = 1)	0（0.00）	1（100.00）		
Live in urban areas				
Live in urban areas = 0(n = 411)	110（26.76）	301（73.24）	11.369	0.001
Live in urban areas = 1(n = 1199)	430（35.86）	769（64.14）		
Overweight/obesity				
Overweight/obesity = 0(n = 1119)	382（34.14）	737（65.86）	0.587	0.444
Overweight/obesity = 1(n = 491)	158（32.18）	333（67.82）		
Relatives and friends with				

noncommunicable diseases				
Relatives and friends with noncommunicable diseases = 0(n = 1143)	361（31.58）	782（68.42）	6.769	0.009
Relatives and friends with noncommunicable diseases = 1(n = 467)	179（38.33）	288（61.67）		
Salt knowledge level				
Salt knowledge level = 1(n = 303)	91（30.03）	212（69.97）		
Salt knowledge level = 2(n = 581)	186（32.01）	395（67.99）	5.357	0.147
Salt knowledge level = 3(n = 610)	217（35.57）	393（64.43）		
Salt knowledge level = 4(n = 116)	46（39.66）	70（60.34）		
Having awareness of hypertension				
Having awareness of hypertension = 0(n =113)	15（13.27）	98（86.73）	22.392	0.000
Having awareness of hypertension = 1(n = 1497)	525（35.07）	972（64.93）		
Positive attitudes towards a low-salt diet				
Positive attitudes towards a low-salt diet = 0(n = 560)	179（31.96）	381（68.04）	0.957	0.328
Positive attitudes towards a low-salt diet =1(n = 1050)	361（34.38）	689（65.62）		
Taste preference for salty foods				
Taste preference for salty foods = 0(n =1336)	477（35.70）	859（64.30）	16.480	0.000
Taste preference for salty foods = 1(n = 274)	63（22.99）	211（77.01）		
Participation in nutrition education and training program on sodium reduction				
Participation in nutrition education and training program	407（33.31）	815（66.69）	0.125	0.724

on sodium reduction = 0(n = 1222)				
Participation in nutrition education and training program on sodium reduction = 1(n = 388)	133 (34.28)	255 (65.72)		
Community-based education on salt reduction availability				
Community-based education on salt reduction availability = 0(n = 1158)	377 (32.56)	781 (67.44)	1.793	0.181
Community-based education on salt reduction availability = 1(n = 452)	163 (36.06)	289 (63.94)		
Salt intake increase by food advertising				
Salt intake increase by food advertising = 0(n = 1591)	537 (33.75)	1054 (66.25)	2.718	0.099
Salt intake increase by food advertising = 1(n = 19)	3 (15.79)	16 (84.21)		

Source: Author' s own calculations.

Table 4. Chi-Square Test Results of Subgroups from Adoption of Less Pickled Food Intake.

Subgroups	**Less Pickled Food Intake =1[n (%)]**	**Less Pickled Food Intake =0[n (%)]**	**Chi-Square**	***p-value***
Male				
Male = 0(n = 835)	304 (36.41)	531 (63.59)	0.473	0.492
Male = 1(n = 775)	295 (38.06)	480 (61.94)		
Age				
Age = 1(n = 278)	64 (23.02)	214 (76.98)		
Age = 2(n = 381)	131 (34.38)	250 (65.62)		
Age = 3(n = 364)	157 (43.13)	207 (56.87)	36.926	0.000
Age = 4(n = 323)	133 (41.18)	190 (58.82)		
Age = 5(n = 264)	114 (43.18)	150 (56.82)		
Education				
Education = 1(n = 19)	4 (21.05)	15 (78.95)		
Education = 2(n = 101)	26 (25.74)	75 (74.26)		
Education = 3(n = 341)	115 (33.72)	226 (66.28)	14.138	0.007
Education = 4(n = 1063)	414 (38.95)	649 (61.05)		
Education = 5(n = 86)	40 (46.51)	46 (53.49)		
Employment				
Employment = 0(n = 656)	213 (32.47)	443 (67.53)	10.626	0.001

Employment = 1(n = 954)	386 (40.46)	568 (59.54)		
Individual annual income				
Individual annual income = 1(n = 369)	100 (27.10)	269 (72.90)		
Individual annual income = 2(n = 66)	19 (28.79)	47 (71.21)		
Individual annual income = 3(n = 73)	31 (42.47)	42 (57.53)		
Individual annual income = 4(n = 111)	38 (34.23)	73 (65.77)		
Individual annual income = 5(n = 164)	57 (34.76)	107 (65.24)		
Individual annual income = 6(n = 296)	125 (42.23)	171 (57.77)		
Individual annual income = 7(n = 187)	75 (40.11)	112 (59.89)	42.118	0.000
Individual annual income = 8(n = 171)	86 (50.29)	85 (49.71)		
Individual annual income = 9(n = 91)	40 (43.96)	51 (56.04)		
Individual annual income = 10(n = 47)	17 (36.17)	30 (63.83)		
Individual annual income = 11(n = 16)	7 (43.75)	9 (56.25)		
Individual annual income = 12(n = 13)	4 (30.77)	9 (69.23)		
Individual annual income = 13(n = 5)	0 (0.00)	5 (100.00)		
Individual annual income = 14(n = 1)	0 (0.00)	1 (100.00)		
Live in urban areas				
Live in urban areas = 0(n = 411)	120 (29.20)	291 (70.80)	15.148	0.000
Live in urban areas = 1(n = 1199)	479 (39.95)	720 (60.05)		
Self-rated health				
Self-rated health = 1(n = 6)	2 (33.33)	4 (66.67)		
Self-rated health = 2(n = 58)	21 (36.21)	37 (63.79)		
Self-rated health = 3(n = 619)	205 (33.12)	414 (66.88)	7.685	0.104
Self-rated health = 4(n = 666)	268 (40.24)	398 (59.76)		
Self-rated health = 5(n = 261)	103 (39.46)	158 (60.54)		
Overweight/obesity				

Overweight/obesity = 0(n = 1119)	428 (38.25)	691 (61.75)	1.710	0.191
Overweight/obesity = 1(n = 491)	171 (34.83)	320 (65.17)		
Relatives and friends with noncommunicable diseases				
Relatives and friends with noncommunicable diseases = 0(n = 1143)	412 (36.05)	731 (63.95)	2.268	0.132
Relatives and friends with noncommunicable diseases = 1(n = 467)	187 (40.04)	280 (59.96)		
Salt knowledge level				
Salt knowledge level = 1(n = 303)	101 (33.33)	202 (66.67)		
Salt knowledge level = 2(n = 581)	209 (35.97)	372 (64.03)		
Salt knowledge level = 3(n = 610)	251 (41.15)	359 (58.85)	7.362	0.061
Salt knowledge level = 4(n = 116)	38 (32.76)	78 (67.24)		
Having awareness of hypertension				
Having awareness of hypertension = 0(n = 113)	25 (22.12)	88 (77.88)	11.831	0.001
Having awareness of hypertension = 1(n = 1497)	574 (38.34)	923 (61.66)		
Positive attitudes towards a low-salt diet				
Positive attitudes towards a low-salt diet = 0(n = 560)	189 (33.75)	371 (66.25)	4.387	0.036
Positive attitudes towards a low-salt diet = 1(n = 1050)	410 (39.05)	640 (60.95)		
Taste preference for salty foods				
Taste preference for salty foods = 0(n = 1336)	514 (38.47)	822 (61.53)	5.403	0.020
Taste preference for salty foods = 1(n = 274)	85 (31.02)	189 (68.98)		

Participation in nutrition education and training program on sodium reduction				
Participation in nutrition education and training program on sodium reduction = 0(*n* = 1222)	461 (37.73)	761 (62.27)	0.587	0.444
Participation in nutrition education and training program on sodium reduction = 1(*n* = 388)	138 (35.57)	250 (64.43)		
Community-based education on salt reduction availability				
Community-based education on salt reduction availability = 0(*n* = 1158)	436 (37.65)	722 (62.35)	0.351	0.553
Community-based education on salt reduction availability = 1(*n* = 452)	163 (36.06)	289 (63.94)		
Salt intake increase by food advertising				
Salt intake increase by food advertising = 0(*n* = 1591)	591 (37.15)	1000 (62.85)	0.198	0.657
Salt intake increase by food advertising = 1(*n* = 19)	8 (42.11)	11 (57.89)		

Source: Author's own calculations.

Supplementary Materials C: Estimation Details

1. The Estimation Details of Lasso Logistic Regression

As shown from Figure 1 to Figure 4, the Optimal λ and optimal numbers of variables selected were estimated by using 10-fold cross-validation in lasso logistic regression model of each practice.

The Optimal λ were 0.040, 0.043, 0.031,0.027, while optimal numbers of variables selected were 3,2,4,6 in the estimation results for adoption of labeled salt information usage, salt-restriction spoon usage, less condiment added in home cooking, less pickled food intake ,respectively.

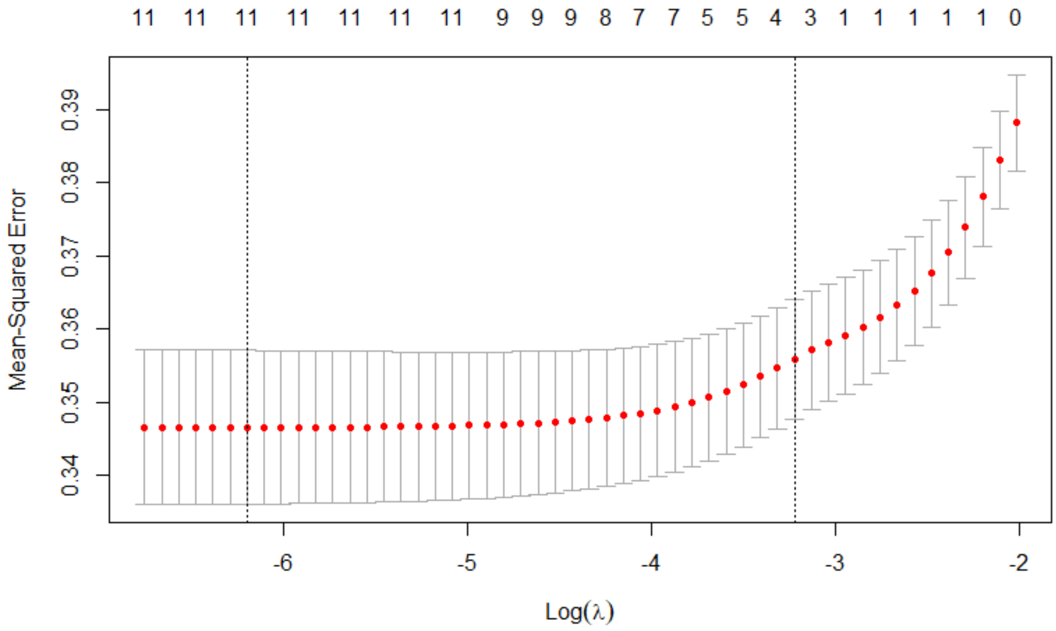

Figure S1. The Optimal λ and optimal numbers of variables selected of the lasso logistic regression model of adoption of labeled salt information usage. Note: X-axis on top: amounts of variables selected; X-axis below: log(λ); Y-axis on the left: Mean-Squared Error

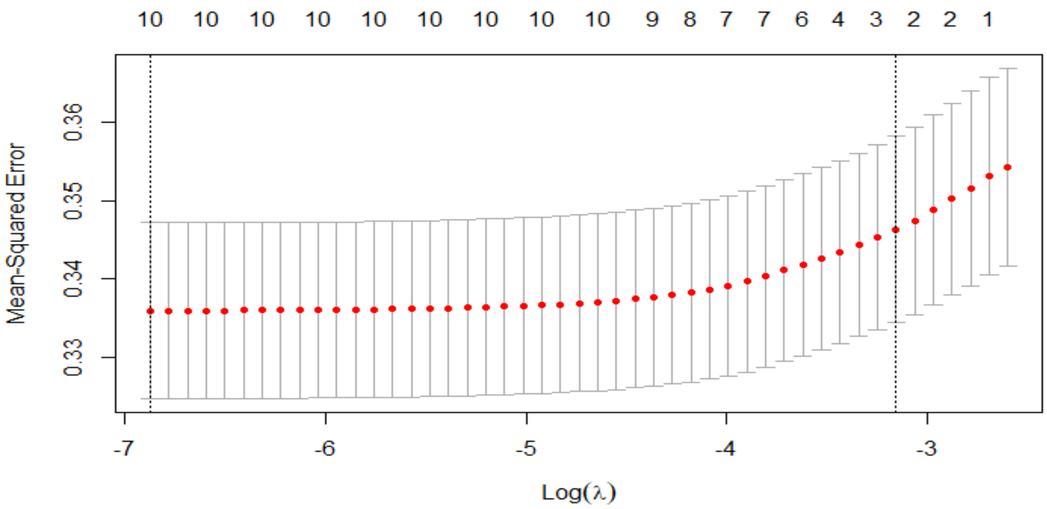

Figure S2. The Optimal λ and optimal numbers of variables selected of the lasso logistic regression model of adoption of salt-restriction spoon usage. Note: X-axis on top: amounts of variables selected; X-axis below: log(λ); Y-axis on the left: Mean-Squared Error.

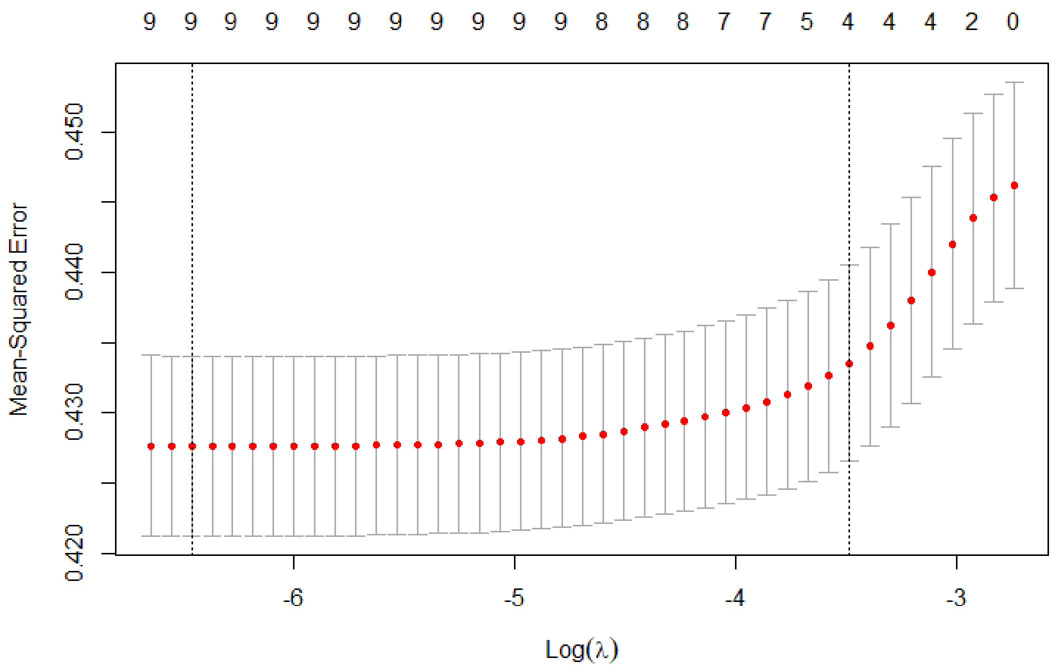

Figure S3. The Optimal λ and optimal numbers of variables selected of the lasso logistic regression model of adoption of less condiment added in home cooking. Note: X-axis on top: amounts of variables selected; X-axis below: log(λ); Y-axis on the left: Mean-Squared Error.

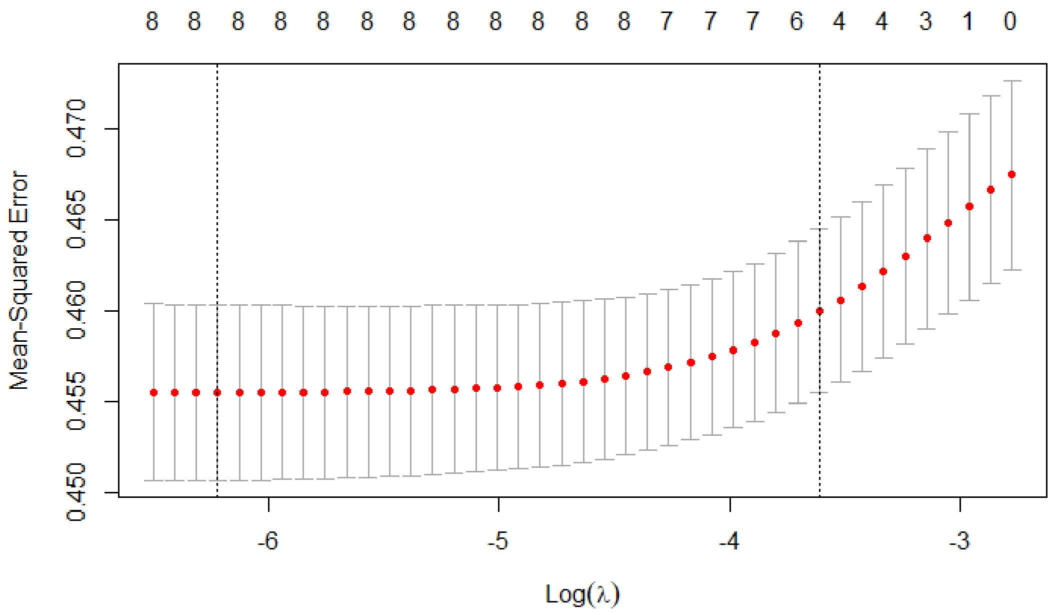

Figure S4. The Optimal λ and optimal numbers of variables selected of the lasso logistic regression model of adoption of less pickled food intake Note: X-axis on top: amounts of variables selected; X-axis below: log(λ); Y-axis on the left: Mean-Squared Error.

2. The Estimation Details of Adaptive Lasso Logistic Regression

As shown from Figure 5 to Figure 12, the move path of the variables were estimated by means of least angle regression (LAR) in adaptive lasso logistic model of each practice.

```
Sequence of LASSO moves:
     x16 x11 x19 x12 x5 x4 x2 x20 x6 x14 x3
Var    9   6  10   7  4  3  1  11  5   8  2
Step   1   2   3   4  5  6  7   8  9  10 11
```

Figure S5. The move path of the adaptive lasso logistic regression model of adoption of labeled salt information usage.

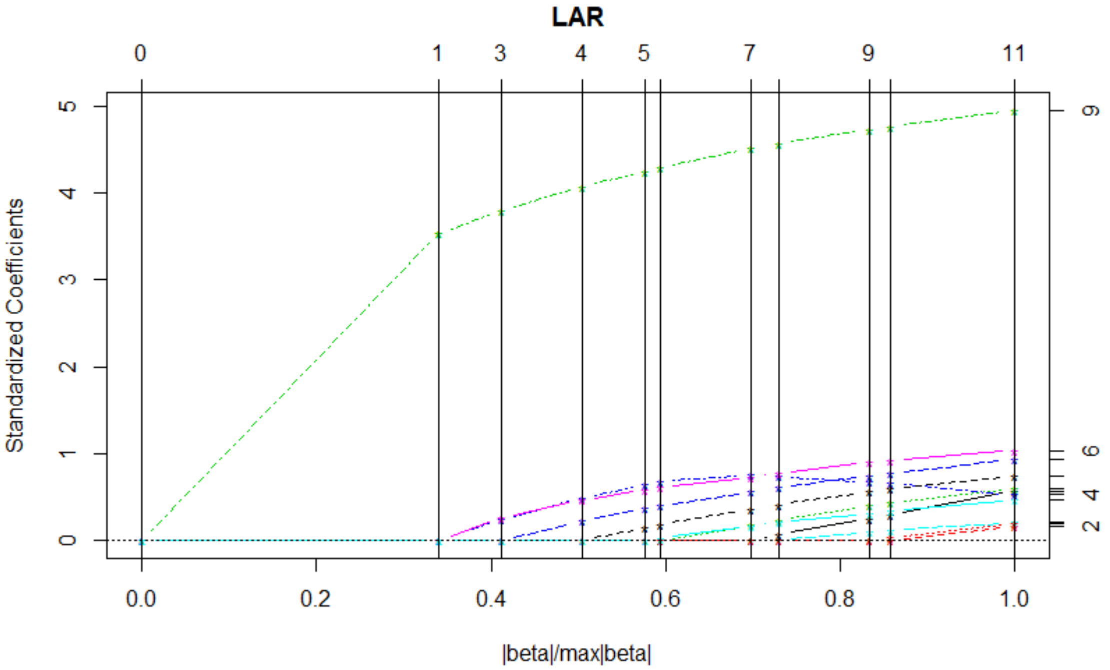

Figure S6. The move path diagram of the adaptive lasso logistic regression model of adoption of labeled salt information usage. Note: X-axis on top: amounts of variables selected; X-axis below: adjusted parameters; Y-axis on the left: standardized coefficients; Y-axis on the right: the code of the corresponding variable

```
Sequence of LASSO moves:
     x13 x18 x10 x12 x17 x11 x20 x19 x2 x6
Var    6   8   3   5   7   4  10   9  1  2
Step   1   2   3   4   5   6   7   8  9 10
```

Figure S7. The move path of the adaptive lasso logistic regression model of adoption of salt restriction spoon usage.

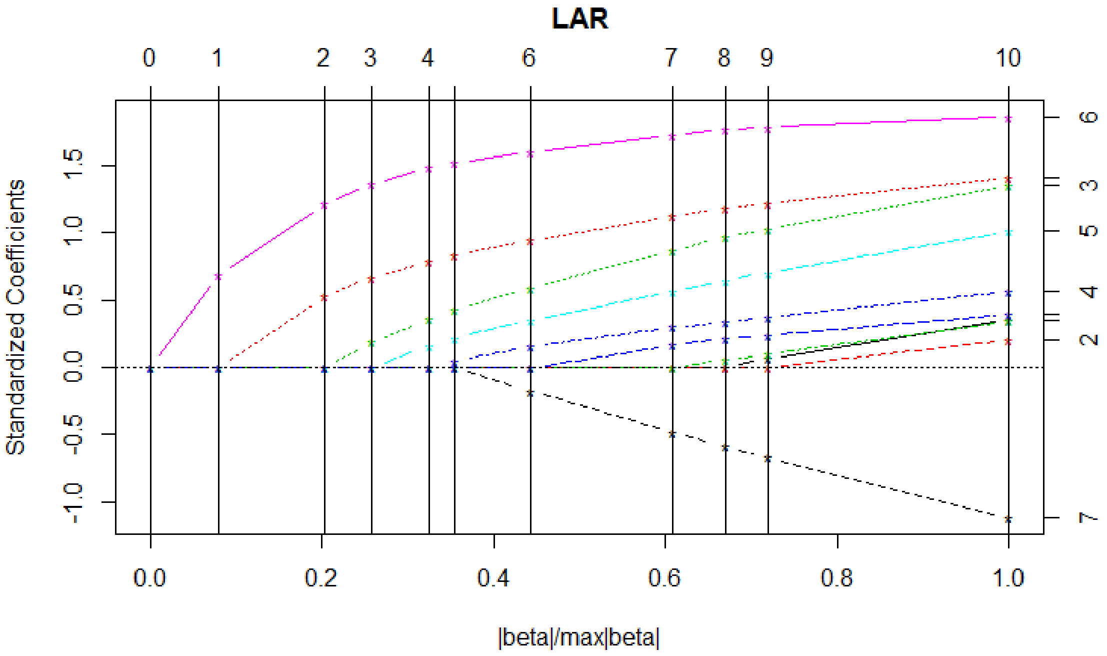

Figure S8. The move path diagram of the adaptive lasso logistic regression model of adoption of salt-restriction spoon usage. Note: X-axis on top: amounts of variables selected; X-axis below: adjusted parameters; Y-axis on the left: standardized coefficients; Y-axis on the right: the code of the corresponding variable.

```
Sequence of LASSO moves:
     x2 x12 x17 x3 x10 x21 x4 x5 x6
Var   1   7   8  2   6   9  3  4  5
Step  1   2   3  4   5   6  7  8  9
```

Figure S9. The move path of the adaptive lasso logistic regression model of adoption of less condiment added in home cooking.

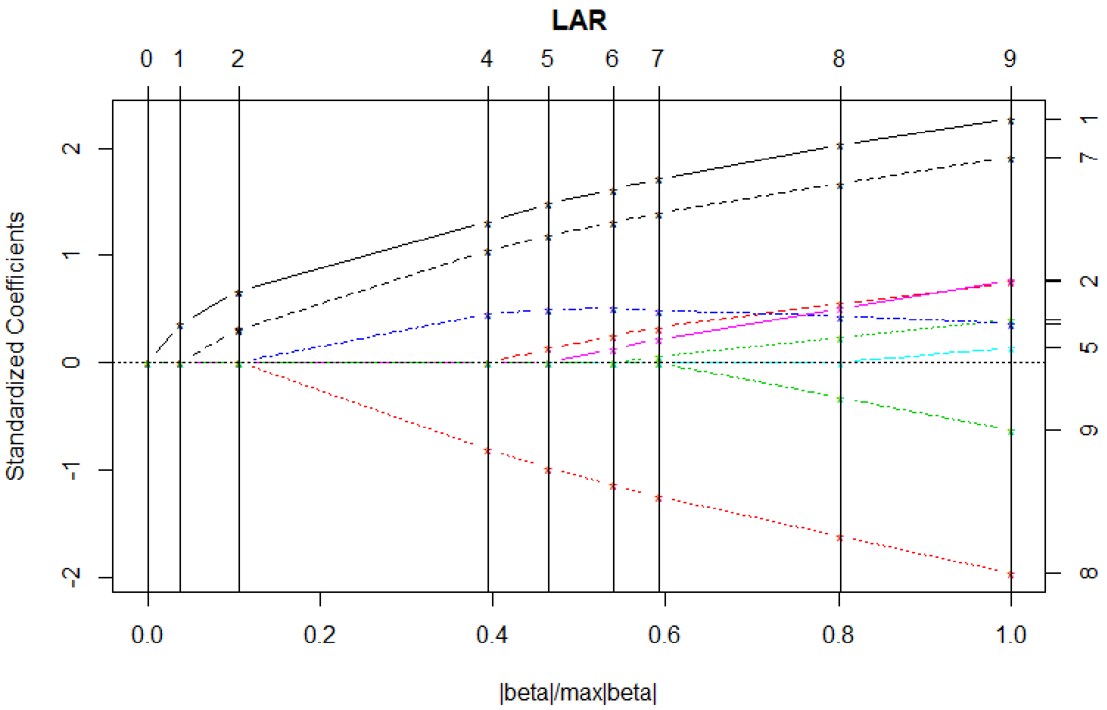

Figure S10. The move path diagram of the adaptive lasso logistic regression model of adoption of less condiment added in home cooking. Note: X-axis on top: amounts of variables selected; X-axis below: adjusted parameters; Y-axis on the left: standardized coefficients;Y-axis on the right: the code of the corresponding variable.

```
Sequence of LASSO moves:
     x2 x3 x12 x17 x14 x6 x4 x5
Var   1  2   6   8   7  5  3  4
Step  1  2   3   4   5  6  7  8
```

Figure S11. The move path of the adaptive lasso logistic regression model of adoption of less pickled food intake.

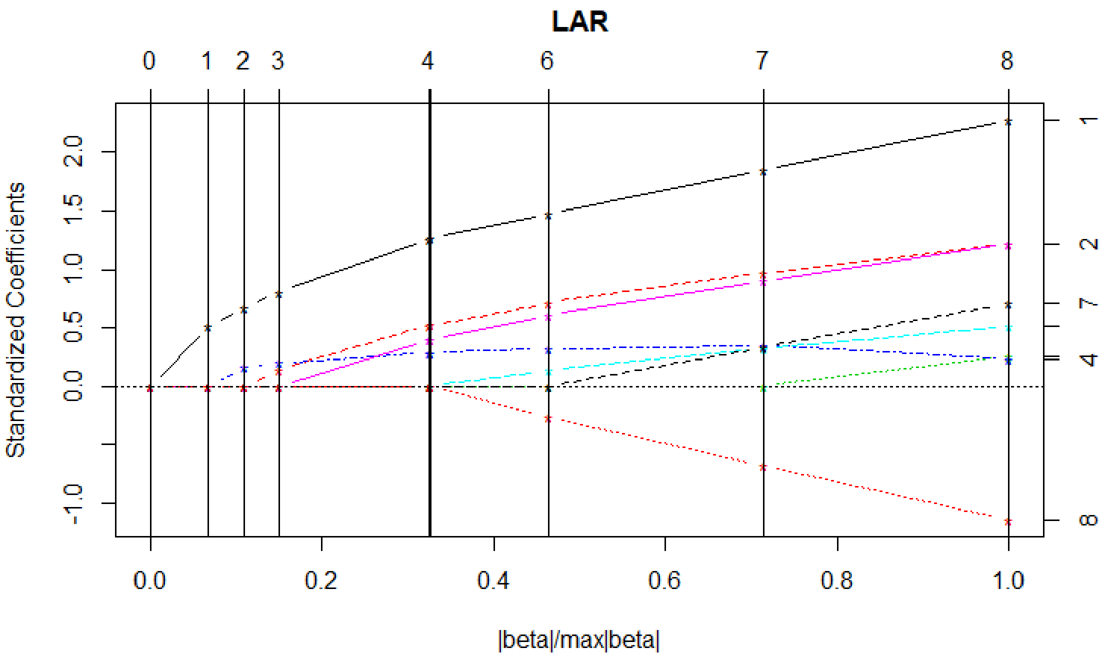

Figure S12. The move path diagram of the adaptive lasso logistic regression model of adoption of less pickled food intake. Note: X-axis on top: amounts of variables selected; X-axis below: adjusted parameters; Y-axis on the left: standardized coefficients; Y-axis on the right: the code of the corresponding variable.

Supplementary Materials D: The Online Salt Knowledge Questionnaire.

Table. The Online Salt Knowledge Questionnaire.

1.What do you know the China dietary recommendations below?	1.1 What is the relationship between salt and sodium? (Salt contains sodium)<br>1.2 How many grams of salt is equivalent to one standard teaspoon of salt? (2 g)<br>1.3 How many sodium per 100 g or 100 ml contain in the pre-packaged food<br>when considered as ‘low in salt’? (120 mg sodium per 100 g or 100 ml)<br>1.4 What is the maximum recommended daily amount of salt for an adult in China? (6g)
2.Which conditions below might be associated with high salt intakes?	2.1 High blood pressure(Yes)<br>2.2 High blood sugar<br>2.3 Stroke(Yes)

	2.4 Kidney disease(Yes)<br>2.5 Osteoporosis
3.What is salt content of commonly eaten foods below?	3.1 White bread (Medium)<br>3.2 Bacon (High)<br>3.3Boiled white rice (Low)<br>3.4Uncooked beef steak (Low)<br>3.5Frozen mix vegetables (Low)<br>3.6Corn flakes (Medium)<br>3.7 Fresh food(Low)<br>3.8Fast foods (High)
4.Which statements below are misconceptions?	4.1Sea salt is better than table salt(No)<br>4.2Drinking more water can neutralize salt in my diet(No)<br>4.3Cutting down on salt causes leg cramps(No)

The questionnaire is from Sarmugam et al.(2014) ,but some questions has been revised and removed in light of series of expert panel discussion Correct answers are in parentheses after each item.
